# MicroRNA-424/E2F6 feedback loop modulates cell invasion, migration and EMT in endometrial carcinoma

**DOI:** 10.18632/oncotarget.23218

**Published:** 2017-12-13

**Authors:** Zheng Lu, Zhou Nian, Zhang Jingjing, Luo Tao, Li Quan

**Affiliations:** ^1^ Department of Gynaecology, The First Affiliated Hospital of Zunyi Medical College, Zunyi, Guizhou 563000, P.R. China

**Keywords:** miR-424, E2F6, endometrial carcinoma

## Abstract

Our previous study explored the roles of microRNA-424 (miR-424) in the development of endometrial carcinoma (EC) and analyzed the miR-424/E2F7 axis in EC cell growth. In this study, we investigated the status of miR-424 in human endometrial cancer tissues, which were collected from a cohort of Zunyi patients. We found that the expression level of miR-424 was associated with clinical tumor stage, cell differentiation, lymph node metastasis and cell migration ability. Cell function experiments demonstrated that miR-424 overexpression suppressed the invasion and migration abilities of endometrial carcinoma cells *in vitro*. Bioinformatic predictions and dual-luciferase reporter assays suggested E2F6 as a possible target of miR-424. RT-PCR and western blot assays demonstrated that miR-424 transfection reduced the expression level of E2F6, while inhibiting miR-424 with ASO-miR-424 (antisense oligonucleotides of miR-424) increased the expression level of E2F6. Cell function experiments indicated that E2F6 transfection rescued the EC cell phenotype induced by miR-424. In addition, we also found that E2F6 negatively regulated miR-424 expression in EC cells. In summary, our results demonstrated that the miR-424/E2F6 feedback loop modulates cell invasion, migration and EMT in EC and that the miR-424/E2Fs regulation network may serve as a new and potentially important therapeutic target in EC.

## INTRODUCTION

Endometrial carcinoma (EC) is the fourth most frequent tumor malignancy in women in the developed world, following breast, colorectal and lung cancer, and recently, its incidence has increased worldwide [1]. In 2014, there were 52,630 new suspected cases of EC with an estimated 8,590 deaths in the USA [2]. However, in 2016, 60,050 women were diagnosed with EC, and 10,470 women died from this disease [3]. Despite most cases being diagnosed in the early stages of disease, 28% of patients still have regional or distant metastasis. EC has a low 5-year survival rate at the incurable stages and a high rate of recurrence and metastasis despite recent advances in therapeutic strategies [4]. Although targeted molecular therapies provide useful future strategies for controlling endometrial malignancies [5], they are still largely undiscovered. Thus, it is important to identify molecular mechanisms involved in the development and progression of EC and to discover novel drug targets.

MicroRNAs (miRNAs) are a class of small, endogenous noncoding RNAs approximately 20-25 nucleotides in length that play key roles in biological processes relevant to cancer, such as tumor angiogenesis, proliferation, cell differentiation, apoptosis and metastasis [6–9]. MiRNAs have been found to regulate posttranscriptional gene expression by binding to the 3′-untranslated region (3′-UTR) of target mRNAs [9], and up to 30% of human genes are regulated by miRNAs [10]. Accordingly, an increasing number of studies have demonstrated that various miRNAs are involved in the progression and biological processes of EC [11–16]. For example, microRNA-125b inhibited EC invasion by targeting ERBB2 [17]. MiR-106b suppressed EC cell invasion by downregulating TWIST1 expression [18]. A previous study from our and those from other groups have been confirmed that miR-424 is involved in the progression and development of several kinds of tumors, such as bladder cancer, non-small cell lung cancer (NSCLC), gastric cancer and EC [19–21]. However, the precise role of miR-424 in EC tumorigenesis needs to be further understood.

In the present study, we investigated the possible roles of miR-424 in EC invasion, migration and EMT, which may contribute to EC metastasis. We analyzed the potential effects of miR-424 expression on EC cell metastasis using HEC-1A and Ishikawa cells. Furthermore, luciferase reporter assays confirmed that miR-424 downregulated the expression of E2F6 in EC by directly targeting the 3’UTR of E2F6 mRNA. We also found indications that there is a negative feedback loop between miR-424 and E2F6 that participates in EC metastasis.

## RESULTS

### Correlation of miR-424 expression with the pathogenesis and aggressiveness of endometrial carcinoma

**Figure 1:**
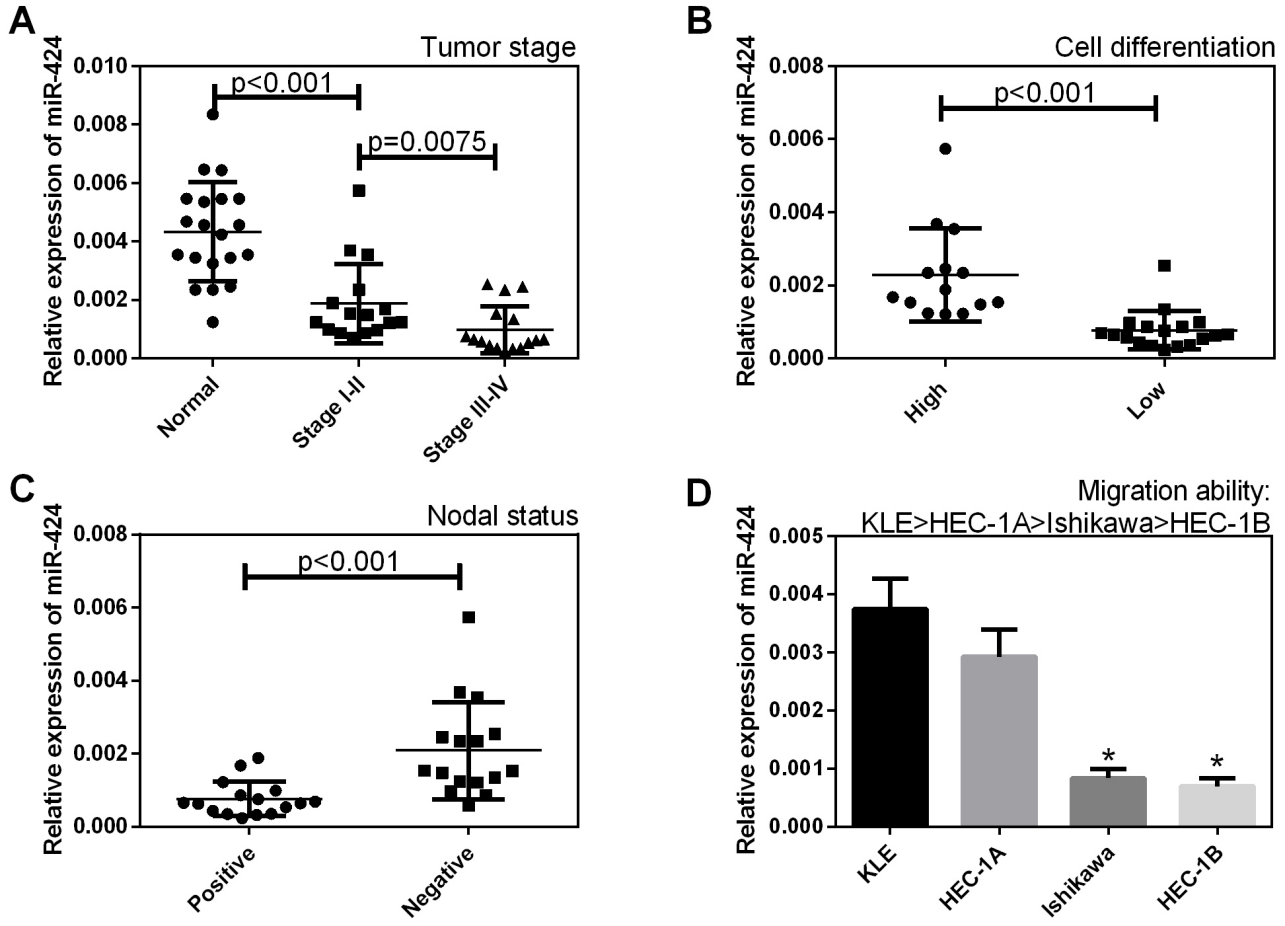
Correlation of miR-424 expression with the pathogenesis and aggressiveness of endometrial carcinoma The expression level of miR-424 was analyzed in endometrial carcinoma samples and normal samples by qRT-PCR. **(A)** The miR-424 expression level was lower in stage I-IV EC tissues than in normal sample tissues, **(B)** in tissues with low levels of cell differentiation than in tissues with high levels of cell differentiation, and **(C)** in lymph nodes with metastasis (+) than in lymph nodes without metastasis (–). **(D)** MiR-424 expression was lower in EC cells with a low migration ability than in those with a high migration ability. All error bars indicate the means ± SD. Experiments were performed in triplicate. ^*^P < 0.05 compared with the control group.

The expression levels of miR-424 in endometrial carcinoma samples and normal samples were analyzed by qRT-PCR. As shown in Figure 1, miR-424 expression levels were lower in stage I-IV EC tissues, in tissues with low levels of cell differentiation and in lymph nodes with metastasis (+) than in normal sample tissues (*P* < 0.05, Figure 1A), in tissues with high levels of cell differentiation (*P* < 0.05, Figure 1B) and in lymph nodes without metastasis (–) (*P* < 0.05, Figure 1C), respectively. In addition, miR-424 expression was lower in EC cells with a low migration ability than in those with a high migration ability (*P* < 0.05, Figure 1D).

### MiR-424 suppresses the invasion and migration of cells from the HEC-1A and Ishikawa endometrial cancer cell lines

**Figure 2:**
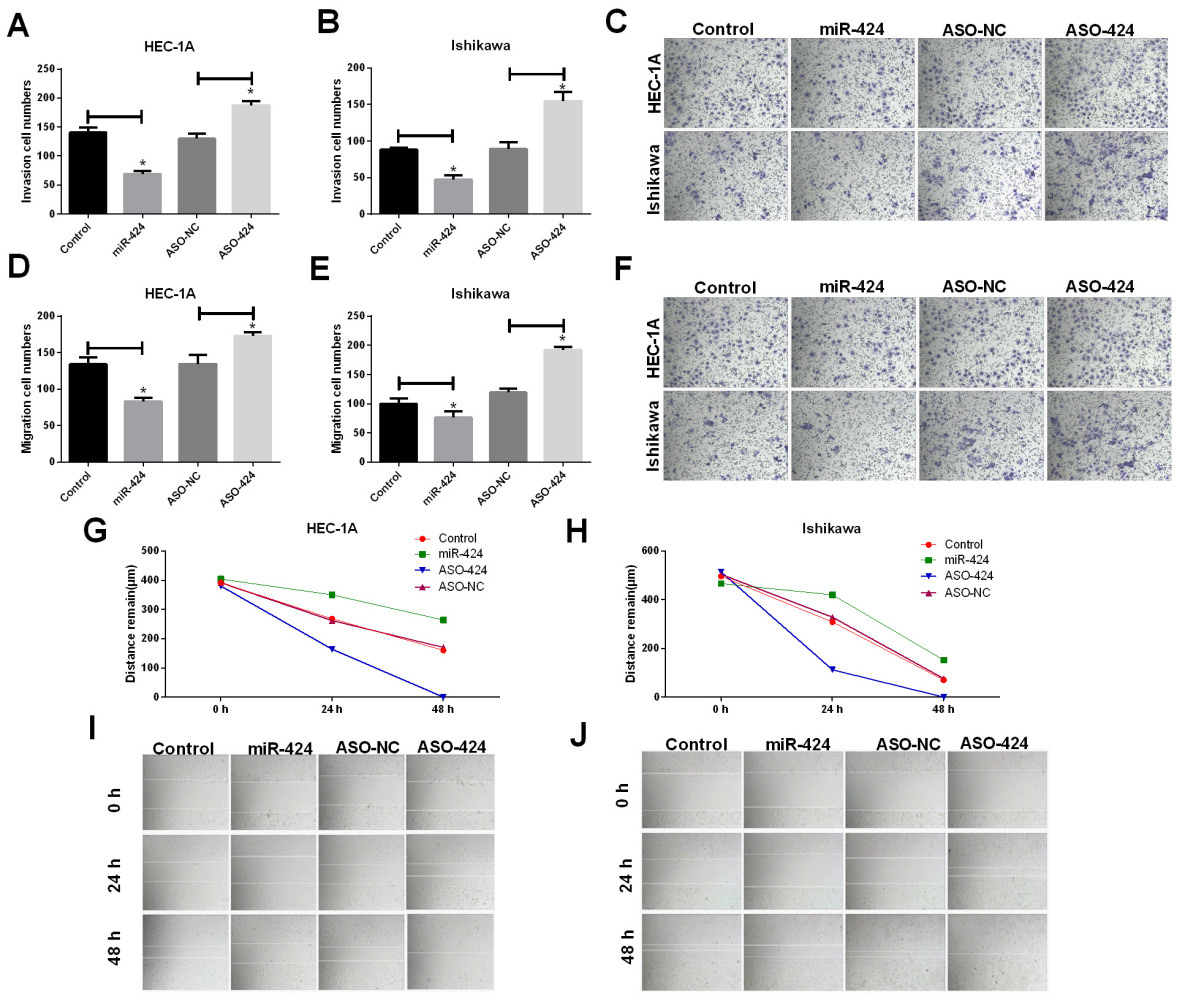
Influence of miR-424 on HEC-1A and Ishikawa cell invasion and migration **(A-C)** The invasion ability of HEC-1A and Ishikawa cells was significantly decreased when cells were transfected with miR-424; however, it was increased when cells were transfected with ASO-424. **(D-F)** The migration ability of HEC-1A and Ishikawa cells was significantly decreased when cells were transfected with miR-424 but was increased when cells were transfected with ASO-424. **(G-J)** The scratch recovery ability of HEC-1A and Ishikawa cells was significantly decreased when cells were transfected with miR-424 but was increased when cells were transfected with ASO-424. All error bars indicate the means ± SD. Experiments were performed in triplicate. ^*^P < 0.05 compared with the control group.

After HEC-1A and Ishikawa cells were transfected with NC, hsa-miR-424 mimics, ASO-hsa-miR-424 or ASO-negative control (NC), invasion and migration abilities were assessed. The results revealed that miR-424 suppressed cell invasion (Figure 2A–2C), migration (Figure 2D–2F) and scratch repair ability (Figure 2G–2J) in HEC-1A and Ishikawa cells; however, ASO-miR-424 promoted cell invasion, migration and scratch repair in HEC-1A and Ishikawa cells (Figure 2).

### MiR-424 suppresses EMT in HEC-1A and Ishikawa endometrial cancer cell lines

**Figure 3:**
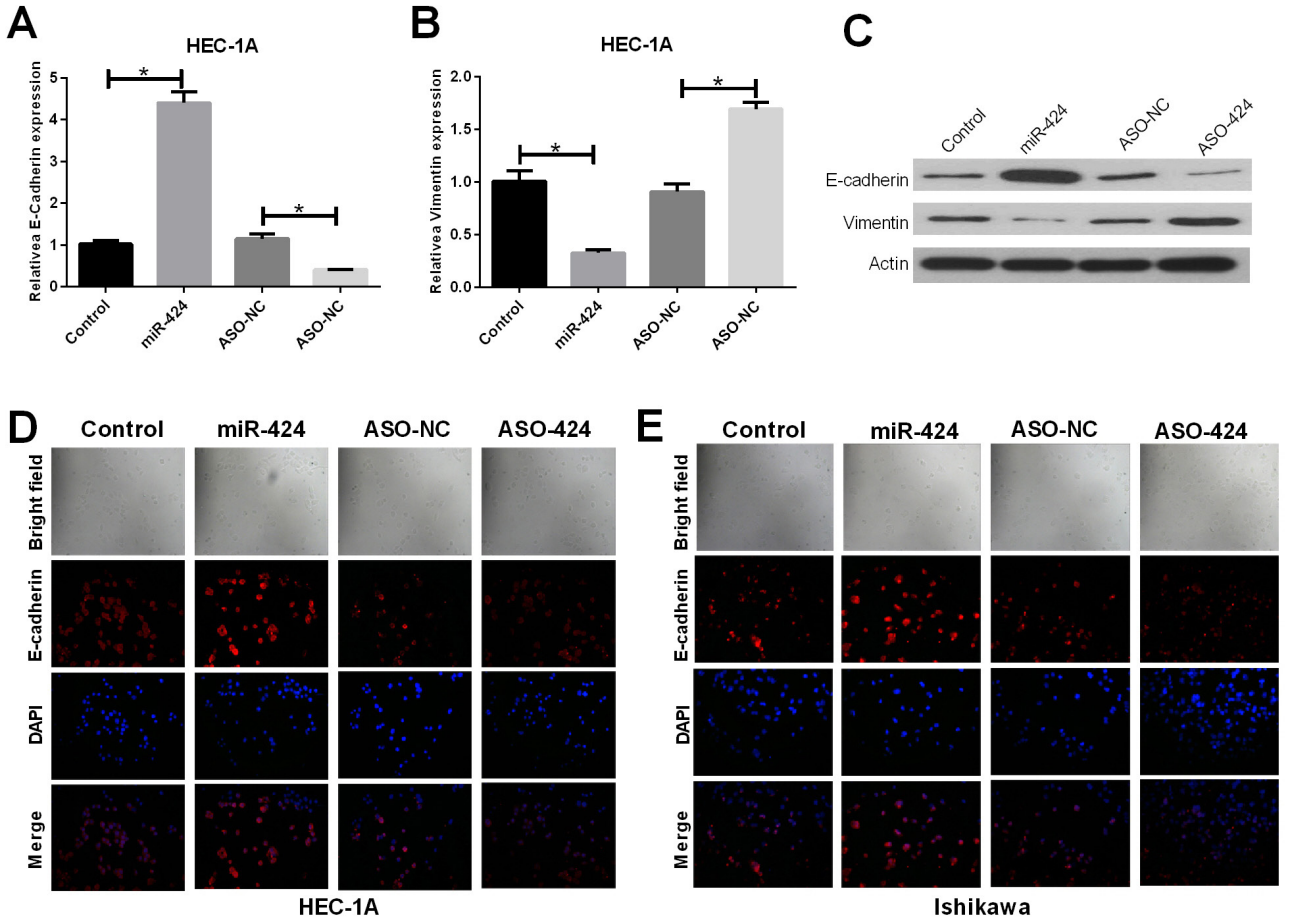
Influence of miR-424 on EMT markers vimentin and E-cadherin **(A-C)** The relative mRNA and protein expression levels of E-cadherin were measured by RT-qPCR and western blot analysis, respectively. **(D-E)** E-cadherin (red) localization and expression were assessed by immunofluorescent analysis in NC, miR-424 mimics, ASO-miR-424 or ASO-negative control (NC) transfected HEC-1A and Ishikawa cells.

RT-PCR and western blot assays showed that E-cadherin was upregulated in miR-424-transfected HEC-1A cells; however, E-cadherin was downregulated in ASO-miR-424-transfected HEC-1A cells. In addition, vimentin was downregulated in miR-424-transfected HEC-1A cells but was upregulated in ASO-miR-424-transfected HEC-1A cells (Figure 3A–3C). Immunofluorescence analysis showed that the E-cadherin protein was overexpressed in miR-424-transfected HEC-1A and Ishikawa cell lines; however, the protein showed low expression in ASO-miR-424-transfected HEC-1A and Ishikawa cell lines. In addition, E-cadherin was distributed mainly in the cytoplasm (Figure 3D, 3E). These results suggest that miR-424 suppresses EMT in the HEC-1A and Ishikawa endometrial cancer cell lines.

### E2F6 gene 3’-UTR carries a putative hsa-miR-424 binding site and is negatively regulated by miR-424

**Figure 4:**
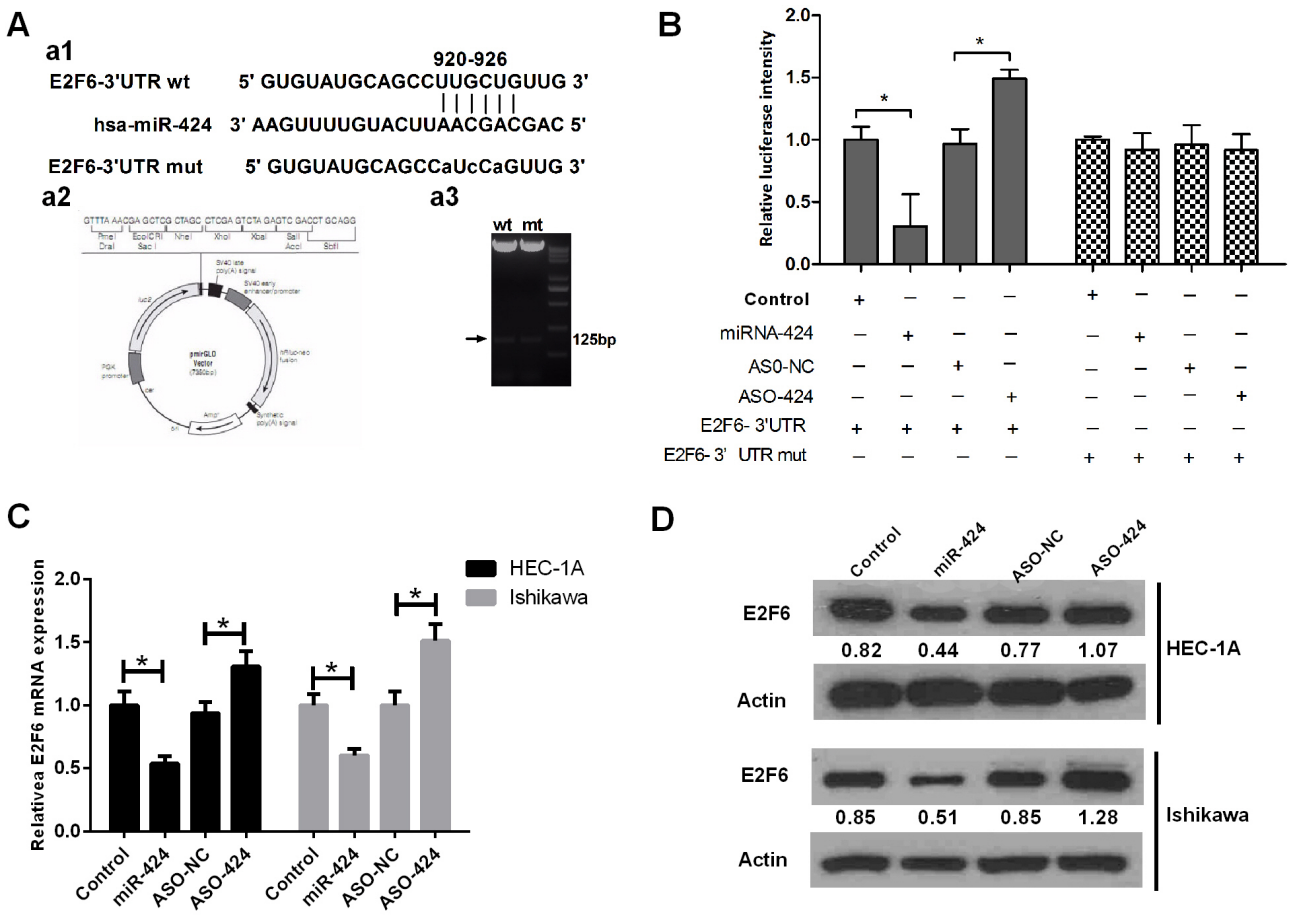
E2F6 gene 3’-UTR carries a putative miR-424 binding site and is negatively regulated by miR-424 **(A)** a1. Bioinformatics analysis predicted that E2F6 is the target gene of hsa-miR-424. a2. Construction of pmirGLO-luciferase reporter vectors (wild-type and mutant). **(B)** Luciferase reporter vectors confirmed that miR-424 can bind directly to the 3’-UTR of E2F6 mRNA. **(C)** The relative E2F6 mRNA expression level is regulated by miR-424. **(D)** The relative E2F6 protein expression level is regulated by miR-424, as determined by western blot analysis. (ASO: antisense oligonucleotides; NC: negative control.) All error bars indicate the means ± SD. Experiments were performed in triplicate. ^*^P < 0.05 compared with the corresponding control group.

Using RNA22 software in our previous study, we predicted that the 3’-UTR of E2F6 mRNA contains a miR-424 binding site. In this study, we cloned the putative binding site (wild-type or mutant) into the pmirGLO plasmid (Figure 4A) and co-transfected this plasmid into cells with NC, miR-424 mimics, ASO-miR-424 or ASO-negative control (NC). We found that the relative luciferase intensity was significantly lower in the cells co-transfected with miR-424 mimics than in those co-transfected with NC. However, the relative luciferase intensity was significantly higher in the cells that were co-transfected with ASO-miR-424 than in the cells co-transfected with ASO-NC (Figure 4B). Additionally, we performed RT-qPCR and western blot analysis to measure the mRNA and protein expression levels of E2F6, respectively. Both RT-qPCR (Figure 4C) and western blot analysis (Figure 4D) revealed that the E2F6 expression levels in HEC-1A and Ishikawa cells were significantly lower in the miR-424 mimics-transfected group than in the NC-transfected group (*P <* 0.05); however, these levels were significantly higher in the ASO-miR-424-transfected group than in the ASO-NC transfected group (*P <* 0.05). These results suggest that hsa-miR-424 binds directly to the 3’-UTR of E2F6 mRNA and inhibits gene expression. These results also indicate that E2F6 is a direct target of miR-424.

### MiR-424 functioned as a tumor suppressor gene by targeting E2F6

**Figure 5:**
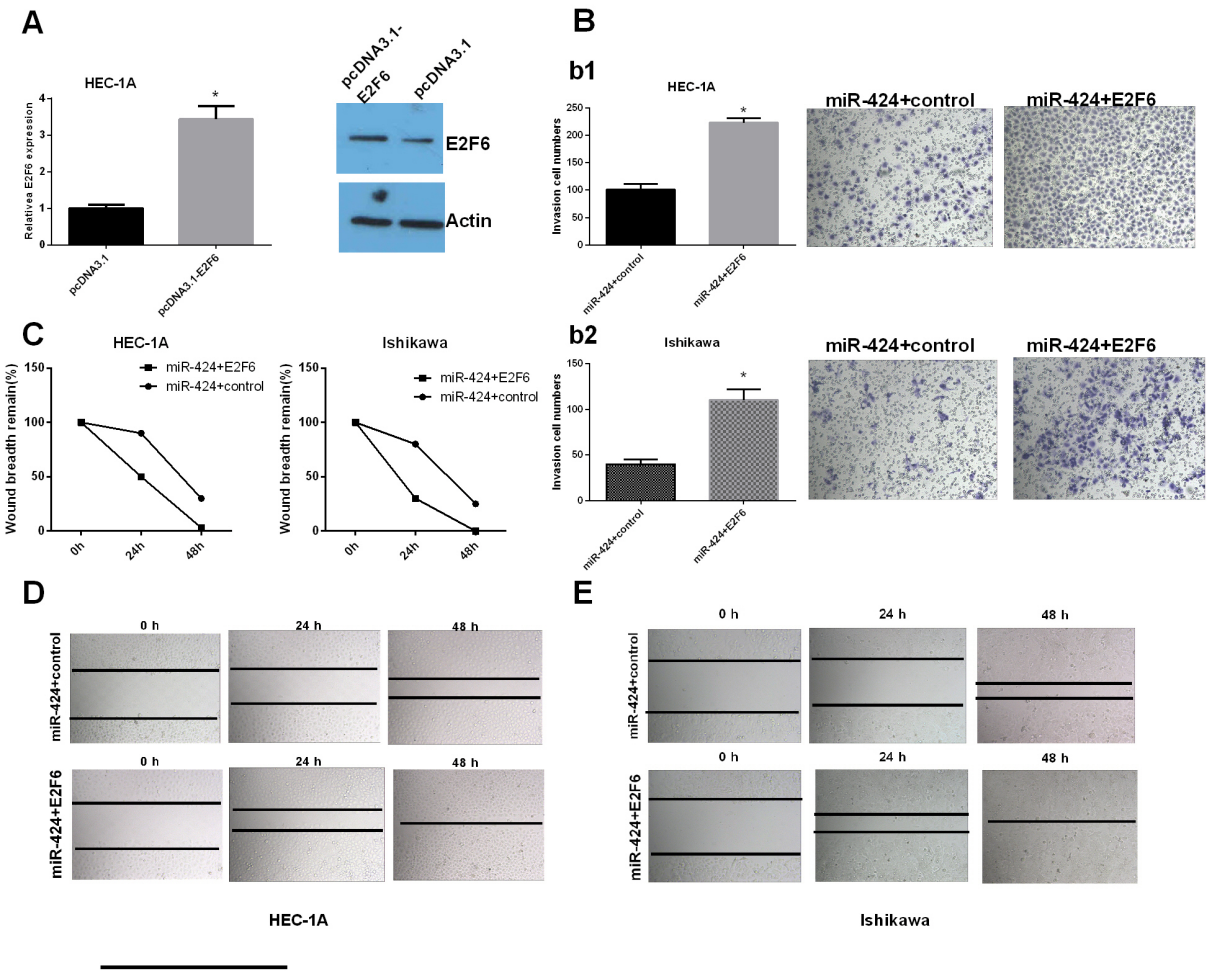
miR-424 functions as a tumor suppressor gene by targeting E2F6 HEC-1A and Ishikawa cells were transfected with miR-424 with/without the E2F6 overexpression plasmid. **(A)** E2F6 relative mRNA and protein expression levels in transfected HEC-1A cells. **(B)** Transwell invasion assay was performed to detect the effect of miR-24-3p and E2F6 on the invasion ability of HEC-1A (b1) and Ishikawa (b2) cells. **(C-E)** Wound healing assays were used to detect the migration capability of transfected cells. All error bars indicate the means ± SD. Experiments were performed in triplicate. ^*^P < 0.05 compared with the corresponding control group.

**Figure 6:**
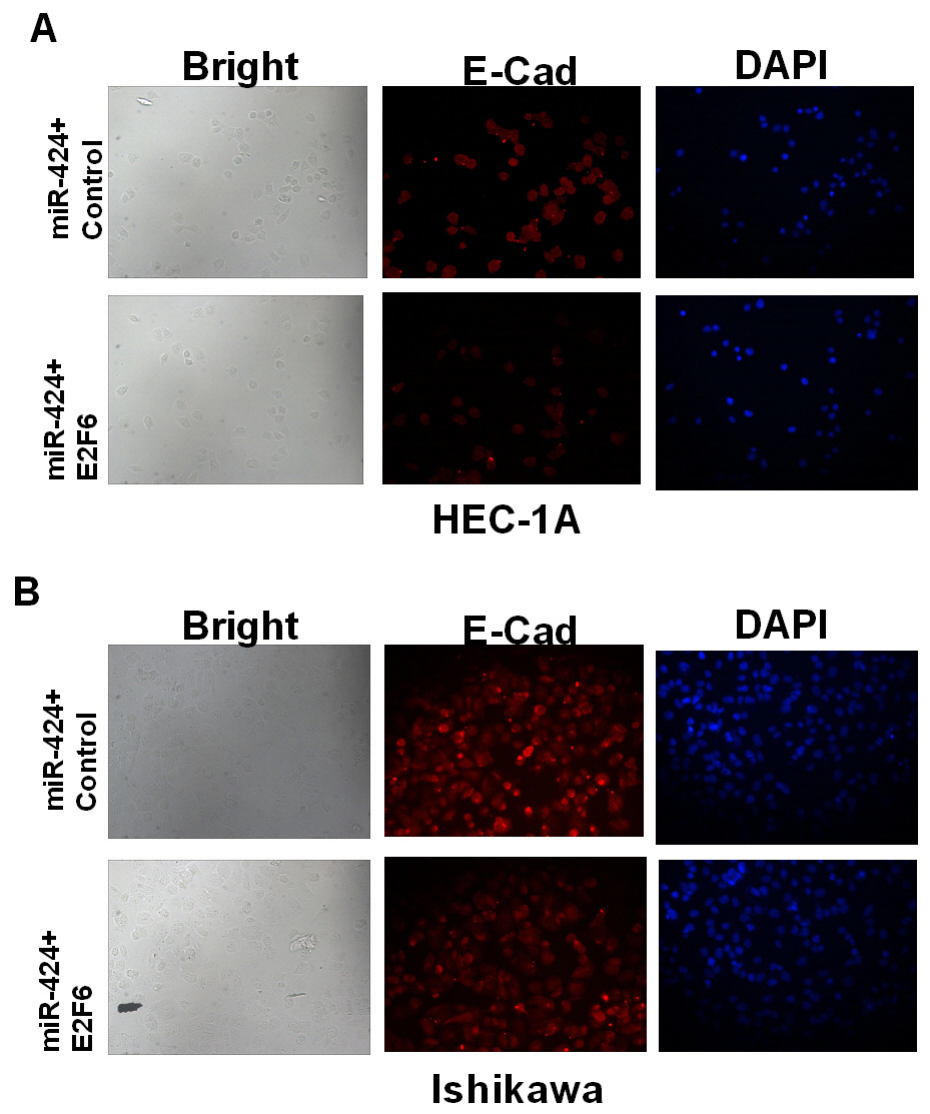
E2F6 reversed the promoting effect of miR-424-mediated EC cell adhesion **(A)** HEC-1A and **(B)** Ishikawa cells were transfected with miR-424 with/without the E2F6 overexpression plasmid. Immunofluorescence staining shows decreased levels of E-cadherin and cell-cell adherence with E2F6 overexpression. DAPI staining of HEC-1A and Ishikawa nuclei.

We further studied whether miR-424 functioned as a tumor suppressor gene by targeting E2F6. Transfection efficiency was determined by qRT-PCR. The relative expression of E2F6 was significantly increased in HEC-1A cells transfected with pcDNA3.1-E2F6 compared with that in the control group (Figure 5A). Cell invasion and wound healing assay results indicated that the overexpression of E2F6 attenuated the miR-424-mediated inhibition of EC cell line (HEC-1A and Ishikawa cells) invasion and migration (*P <* 0.05) (Figure 5B–5E). HEC-1A and Ishikawa cells were transfected with miR-424 with/without an E2F6 overexpression plasmid. Immunofluorescence analysis showed that E-cadherin was highly expressed after the overexpression of miR-424 in both HEC-1A and Ishikawa cells. However, E-cadherin was significantly decreased in HEC-1A and Ishikawa cells transfected with both the miR-424 and E2F6 overexpression plasmid. These results indicated that the overexpression of E2F6 attenuated the accelerated EC cell adhesion mediated by miR-424 (Figure 6A–6B). In summary, we suggest that miR-424 suppresses EC cell metastasis, at least in part, by regulating E2F6 expression.

### E2F6 negatively regulates miR-424 in EC Cells

**Figure 7:**
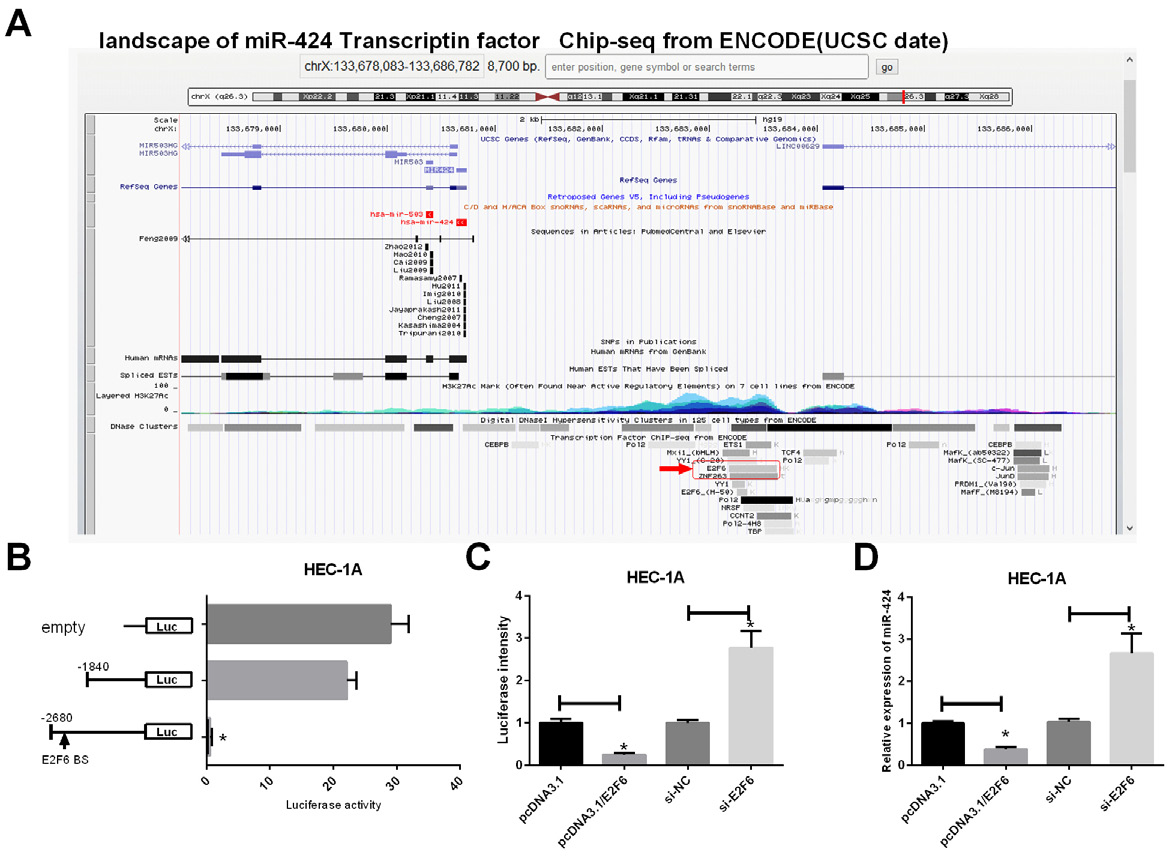
E2F6 regulates miR-424 transcription in EC cells **(A)** Landscape of miR-424 transcription factor from UCSC data. **(B)** Left, schematic presentation of luciferase vectors, a series of deletion mutant vectors. Right, relative luciferase activity constructs. HEC-1A cells were incubated with the E2F6-overexpression plasmid and luciferase reporter. **(C)** Relative luciferase intensity was detected in EC cells after transfection with firefly luciferase constructs and pcDNA3.1, pcDNA3.1/E2F6, si-NC or siE2F6 for 24 h. **(D)** RT-PCR was used to detect the relative expression of miR-424 levels in HEC-1A cells transfected with pcDNA3.1, pcDNA3.1/E2F6, si-NC or siE2F6. All error bars indicate the means ± SD. Experiments were performed in triplicate. ^*^P < 0.05 compared with the corresponding control group.

As shown in Figure 7A, the landscape of the miR-424 transcription factor based on Chip-seq data from ENCODE (UCSC data) indicated the presence of E2F6 binding sites upstream of the miR-424 gene. Next, we constructed a luciferase reporter system (Figure 7B) to co-transfect with E2F6 in HEC-1A cells. The results suggested that E2F6 with the full-length of reporter, which contains the E2F6 binding site upstream of miR-424, inhibits luciferase activity. These results imply that E2F6 can inhibit miR-424 transcription. Additional luciferase assays demonstrated that E2F6 transfection markedly decreased luciferase activity in Luc-wt reporter constructs (*P <* 0.05) (Figure 7C) and decreased the relative expression levels of miR-424 in HEC-1A cells (*P <* 0.05) (Figure 7D), while si-E2F6 increased the luciferase activity in Luc-wt reporter constructs (*P <* 0.05) (Figure 7C) and increased the relative expression levels of miR-424 in HEC-1A cells (*P <* 0.05) (Figure 7D). This result confirms that E2F6 negatively regulates miR-424 in EC cells.

### E2F6 was upregulated in endometrial carcinoma tissues

**Figure 8:**
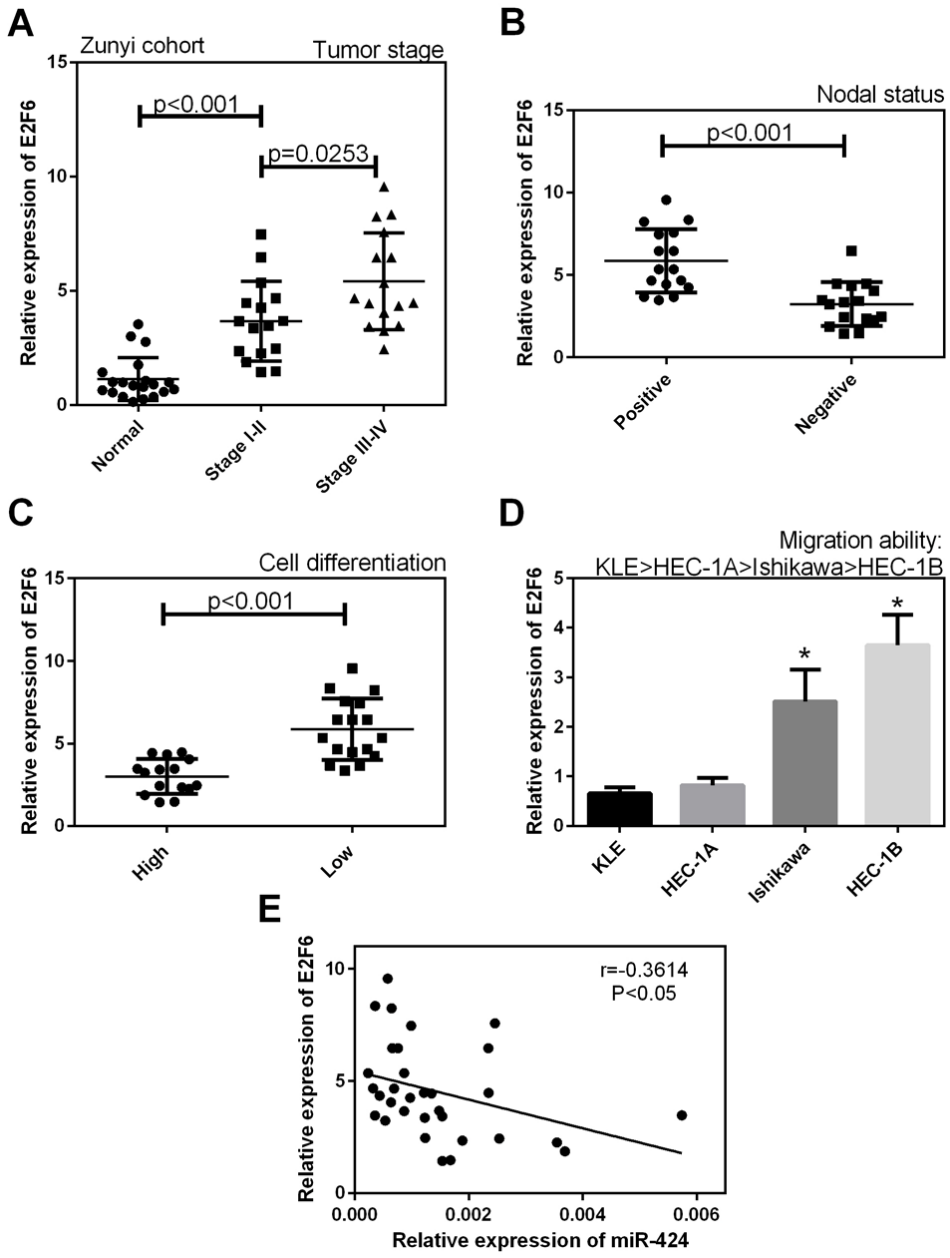
The mRNA expression level of E2F6 in EC tissues in our cohort **(A)** The expression level of E2F6 was higher in stage I-IV EC tissues than in normal samples, **(B)** and in lymph nodes with metastasis (+) than in lymph nodes without metastasis (–). **(C)** E2F6 expression was higher in tissues with low levels of cell differentiation than in tissues with high levels of cell differentiation. **(D)** The expression level of E2F6 was significantly higher in Ishikawa and HEC-1B cell lines with low migration ability than in KLE and HEC-1A cell lines with high migration ability. **(E)** E2F6 expression was negatively correlated with the expression level of miR-424. All error bars indicate the means ± SD. Experiments were performed in triplicate. ^*^P < 0.05 compared with the corresponding control group.

As shown in Figure 8, in the Zunyi cohort, the expression of E2F6 was higher in stage I-IV EC tissues and in lymph nodes with metastasis (+) than in normal tissue samples (*P* < 0.05, Figure 8A) and in lymph nodes without metastasis (–) (*P* < 0.05, Figure 8B), respectively. In addition, E2F6 expression was higher in tissues with low levels of cell differentiation than in tissues with high levels of cell differentiation (*P* < 0.05, Figure 8C). As shown in Figure 8D, the expression of E2F6 was significantly higher in Ishikawa and HEC-1B cell lines with low migration ability than in KLE and HEC-1A cell lines with high migration ability. In addition, we identified that E2F6 expression was negatively correlated with the expression level of miR-424 (Figure 8E).

## DISCUSSION

Studies has demonstrated that miRNAs play crucial roles in the diagnosis, prognostic prediction, and therapy of EC by regulating gene expression, epigenetic dysfunction and carcinogenesis [22]. MiR-424 is a cancer repressor involved in tumor cell proliferation, migration, and invasion [23–25]. Jin et al. identified that miR-424 functions as a tumor suppressor in glioma cells [26]. Wang et al. demonstrated that miR-424 acts as a tumor radiosensitizer by targeting aprataxin in cervical cancer [27]. Zhang et al. reported that miR-424 promotes non-small cell lung cancer metastasis by regulating TNFAIP1 [21]. In present study, we found that the expression levels of miR-424 in EC tissue were lower than in normal tissues and were lower in lymph nodes with metastases than in lymph nodes without metastases. In addition, miR-424 expression was lower in tissue with low levels of cell differentiation than in tissues with high levels of cell differentiation. Patients with lymph node metastases and high levels of cell differentiation have significantly poorer disease-free survival rates than those without metastases [28]. Therefore, miR-424 may be an anti-oncogene in EC. This led us to transfect miR-424 into EC cells to investigate the influence of miR-424 on EC cell tumorigenesis and tumor progression. The results demonstrated that miR-424 suppressed the invasion and migration of endometrial carcinoma cells. However, ASO-miR-424 promoted the invasion and migration of endometrial carcinoma cells.

Myometrial invasion and lymph node metastasis are the main causes of poor prognosis in EC [29, 30] and the epithelial–mesenchymal transition (EMT) has been considered to be a fundamental event in cancer invasion and metastasis [31]. Vimentin and E-cadherin were important biomarkers of epithelial-mesenchymal transition (EMT). However, the role of miR-424 in EC metastasis and EMT remains poorly understood. In our study, the results showed that miR-424 reduced the expression of vimentin and increased the expression of E-cadherin, and ASO-miR-424 upregulated vimentin expression and downregulated E-cadherin expression in EC cell lines. We propose that miR-424 overexpression likely promotes the migration and invasion of EC cells via the epithelial-mesenchymal transition (EMT) pathway.

The mammalian E2F family of transcription factors is crucial in the regulation of cell proliferation, apoptosis and differentiation. Our previous results demonstrated that E2F7 is a target of miR-424 in endometrial cancer [32]. Recently, studies have reported that E2F6 is oncogenic and that its expression is upregulated in prostate and breast cancer [33, 34]. Oberley et al. (23) and Yang et al. (24) reported that E2F6 negatively regulates the tumor-suppressor gene BRCA1 [35, 36]. Zhang et al. identified that miR-424 suppresses estradiol-induced cell proliferation by targeting GPER in endometrial cancer cells [37]. In this study, bioinformatic predictions and dual-luciferase reporter assays found that E2F6 is a possible target of miR-424. Cell function experiments that transfected miR-424 with E2F6 showed an opposite trend to those transfected with miR-424 alone. We determined that miR-424 suppresses the invasion and migration of EC cell lines by targeting the E2F6 gene. The expression levels of E2F6 were higher in stage I-IV EC tissues and in lymph nodes with metastasis (+) than in normal sample tissues and in lymph nodes without metastasis (–), respectively. In addition, E2F6 expression was higher in tissues with low levels of cell differentiation and in Ishikawa and HEC-1B cell lines with a low migration ability than in tissues with high levels of cell differentiation and in KLE and HEC-1A cell lines with a high migration ability, respectively. E2F6 expression was negatively correlated with the expression level of miR-424. In addition, the EMT process was weakened by miR-424 regulation of E2F6 expression. The current study also showed that the decreased expression of E-cadherin due to the overexpression of miR-424 was significantly rescued by E2F6 transfection.

**Figure 9:**
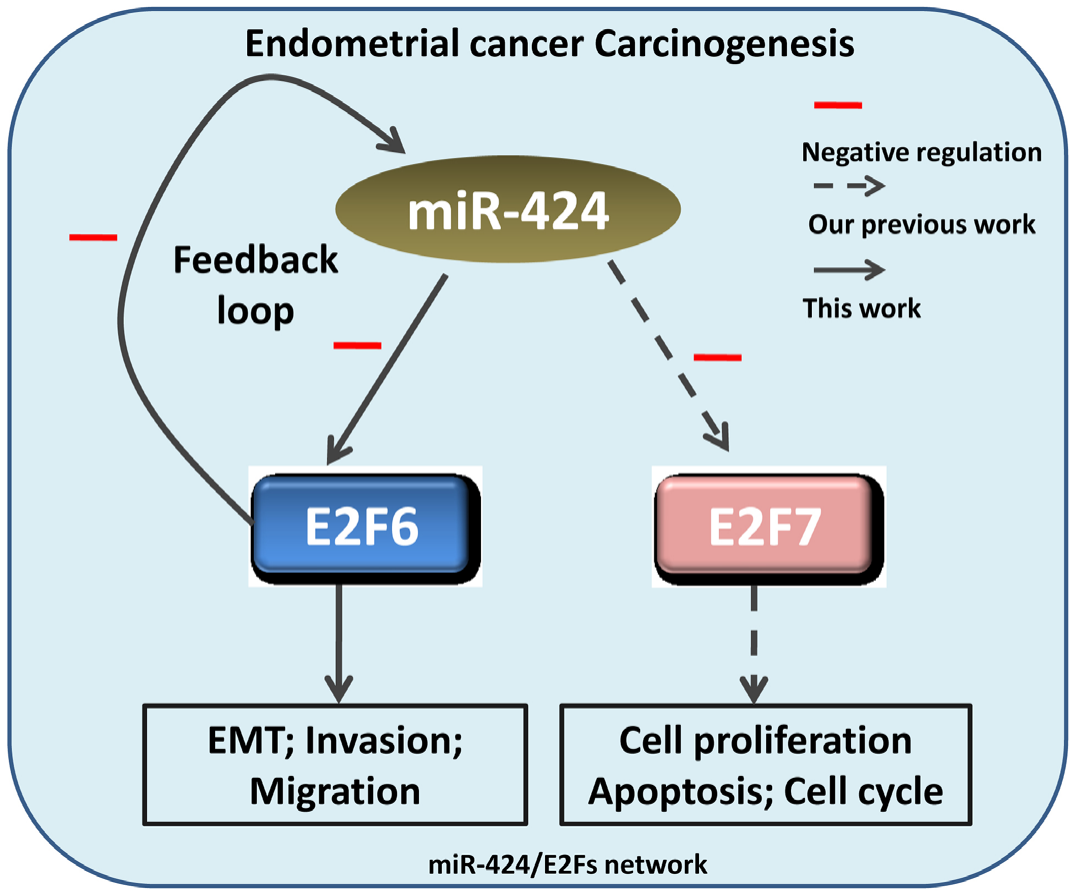
Schematic depiction of the miR-424/E2Fs network in endometrial cancer cell tumorigenesis Our previous study showed that miR-424-mediated the repression of E2F7 in endometrial cancer cells, simultaneously suppressing cell proliferation, increasing cell apoptosis and causing cell cycle arrest. In this study, we identified a feedback loop between miR-424 and E2F6 that modulates cell invasion, migration and EMT in endometrial carcinoma.

In summary, this study indicates that miR-424 acts as a tumor suppressor by targeting E2F6 in endometrial cancer and simultaneously suppresses the progress of cell EMT, invasion and migration. Our previous study showed that miR-424 mediated the repression of E2F7 in endometrial cancer cells, suppressing cell proliferation, increasing cell apoptosis and causing cell cycle arrest. In light of these combined results, we suggest that the miR-424/E2Fs axis may be a potential biomarker and a novel therapeutic target in endometrial cancer (Figure 9).

## MATERIALS AND METHODS

### Tissue samples

Human endometrial cancer tissue and matched adjacent normal tissues from 32 patients with EC were collected from the First Affiliated Hospital of Zunyi Medical College. This study was approved by the ethics committee of the First Affiliated Hospital of Zunyi Medical College, and written informed consent was obtained from all patients. All tissue samples were immediately stored at −80°C after washing with sterile phosphate-buffered saline (PBS).

### Cell culture and transfection

Human EC cells lines, including KLE, HEC-1A, Ishikawa and HEC-1B, were purchased from ATCC (Manassas, VA, USA). The above cells were maintained in Dulbecco’s modified Eagle’s medium (DMEM) (Invitrogen, Carlsbad, CA, USA) supplemented with 10% fetal bovine serum (FBS), 100 IU/ml penicillin and 100 mg/ml streptomycin at 37°C in a humidified atmosphere with 95% air and 5% CO_2_. The negative control mimic, has-miR-424 mimics, antisense oligonucleotides (ASO)-hsa-miR-424, ASO-NC (negative control), pcDNA3.1-E2F6 5-3’-UTR, pcDNA3.1 E2F6 5-3’-UTR and the plasmids were constructed and synthesized by Rui-Jiyin Biotech Co., Ltd. (Tianjin, China). Cells were transfected with the indicated nucleotides or plasmid using Lipofectamine 2000 reagent according to the manufacturer’s instructions.

### RNA extraction and quantitative real-time PCR (qRT-PCR)

Total RNA was extracted from surgical specimens or cell lines using TRIzol reagent (Invitrogen, Carlsbad, CA, USA) according to the manufacturer’s protocol and was reverse transcribed to cDNA by an RT-PCR assay using a miScript II RT Kit (Qiagen, Hilden, Germany). The target genes and controls were analyzed by qRT-PCR, and the reactions were performed on an ABI 7500 system (Applied Biosystems, Carlsbad, CA, USA). All data are presented as the means ± SD of three independent experiments. The relative miRNA expression levels were determined using TaqMan^®^ MicroRNA assays and the comparative 2^-ΔΔCT^ method [38].

### Western blot analysis

Total cell protein was harvested and lysed in RIPA buffer containing protease inhibitors. Protein concentration was determined using a BCA Protein Assay Kit. Equal amounts of total protein were separated on 10% SDS-PAGE gels and transferred onto PVDF membranes (PerkinElmer, Boston, MA). Fat-free milk (5%) was used to block membranes for 2 h at room temperature. After blocking, primary antibodies were incubated with the membrane overnight at 4°C. The membranes were subsequently incubated with HRP-conjugated secondary antibodies and developed using an ECL detection kit according to the manufacturer’s protocol. The primary antibodies in this study were anti-E-cadherin (1:800 dilution, Abcam, ab133597), anti-vimentin (1:800 dilution, Abcam, ab137321), anti-E2F6 (1:800 dilution, Abcam, ab152151), and anti-actin (1:800 dilution, Abcam, ab8226).

### Migration and invasion assays

Matrigel-coated transwell chambers (BD Bioscience, San Jose, CA, USA) were used for the cell migration and invasion assays. Treated HEC-1A or Ishikawa cells (5 × 10^4^) were seeded in the upper compartment and incubated in serum-free media, and the lower compartment was filled with complete medium supplemented with 10% FBS. After incubation for 24 hours at 37°C, cells on the upper surface of the filter were removed using a cotton swab and the migratory and invasive cells on the bottom surface of the filters were fixed in 4% paraformaldehyde and stained by 0.1% crystal violet solution. Four random fields were counted for each group under an Olympus fluorescence microscope. The experiments were performed in triplicate.

### Wound healing assay

Treated HEC-1A or Ishikawa Cells were seeded in 6-well plates and cultured to 80% confluence. Thereafter, small linear wounds were created by removing a line of cells with a disinfected Eppendorf tip. After washing the cell debris with FBS-free medium, images of the wound were captured under a microscope after 0, 24, and 48 h to assess the distance remaining.

### Immunofluorescence

Cells under different conditions were plated onto different 6-well plates and fixed in 4% paraformaldehyde for 20 min, followed by 0.3% Triton X-100 for 10 min. After preincubating with 10% goat serum to block nonspecific binding, cells were incubated with primary antibodies against E-cadherin (1:50, Abcam, ab1416) at 4°C overnight. FITC-conjugated secondary antibody was used for detection and double stained with 4′,6-diamidino-2-phenylindole (DAPI) to visualize the nuclei. Images were observed and captured on an inverted phase/fluorescence microscope.

### Luciferase reporter assay

To confirm that miR-424 can bind to the predicted E2F6 site, we conducted a luciferase reporter assay in the EC cell line. HEC-1A or Ishikawa cells were cultured in a 24-well plate, cotransfected with miR-424, miR-NC, ASO-miR-424, or ASO-NC and either WT or Mut 3′-UTR E2F6. At 48 h after transfection, luciferase activity was measured with a dual-luciferase reporter assay system.

### Statistical analysis

The data were analyzed by Student’s t-test and analysis of variance (ANOVA) using SPSS software (version 19.0, IBM, Chicago, IL, USA). Paired t-tests were used to analyze comparisons between the groups and paired data. Each experiment was repeated at least three times. Data are presented as the means ± standard deviation (SD). Statistical significance was considered when P < 0.05.
